# Biodistribution and Dosimetry of ^177^Lu-tetulomab, a New Radioimmunoconjugate for Treatment of Non-Hodgkin Lymphoma

**DOI:** 10.2174/1874471011306010004

**Published:** 2013-03

**Authors:** Ada H V Repetto-Llamazares, Roy H Larsen, Camilla Mollatt, Michael Lassmann, Jostein Dahle

**Affiliations:** 1 Nordic Nanovector AS, Kjelsåsveien 168 B, 0884 Oslo; 2 Department of Radiation Biology, Institute for Cancer Research, Oslo University Hospital, Montebello, 0310 Oslo, Norway; 3 Sciencons Ltd., Oslo, Norway; 4 Department of Nuclear Medicine, University of Würzburg, Würzburg, Germany

**Keywords:** CD37, Lutetium-177, Non-Hodgkin lymphoma, Radioimmunotherapy.

## Abstract

The biodistribution of the anti-CD37 radioimmunoconjugate ^177^Lu-tetraxetan-tetulomab (^177^Lu-DOTA-HH1) was evaluated. Biodistribution of ^177^Lu-tetraxetan-tetulomab was compared with ^177^Lu-tetraxetan-rituximab and free ^177^Lu in nude mice implanted with Daudi lymphoma xenografts. The data showed that ^177^Lu-tetulomab had a relevant stability and tumor targeting properties in the human lymphoma model. The half-life of ^177^Lu allowed significant tumor to normal tissue ratios to be obtained indicating that ^177^Lu-tetraxetan-tetulomab could be suitable for clinical testing. The biological and effective half-life in blood was higher for ^177^Lu-tetraxetan-tetulomab than for ^177^Lu-tetraxetan-rituximab. The biodistribution of ^177^Lu-tetraxetan-tetulomab did not change significantly when the protein dose was varied from 0.01 to 1 mg/kg. Dosimetry calculations showed that the absorbed radiation doses to normal tissues and tumor in mice were not significantly different for ^177^Lu-tetraxetan-tetuloma b and ^177^Lu-tetraxetan-rituximab. The absorbed radiation doses were extrapolated to human absorbed radiation doses. These extrapolated absorbed radiation doses to normal tissues for ^177^Lu-tetraxetan-tetulomab at an injection of 40 MBq/kg were significantly lower than the absorbed radiation doses for 15 MBq/kg Zevalin, suggesting that higher tumor radiation dose can be reached with ^177^Lu-tetraxetan-tetulomab in the clinic.

## INTRODUCTION

Radioimmunotherapy (RIT) involves targeting a tumor-associated antigen with cytotoxic radiolabeled monoclonal antibodies (mAbs). Radiolabeled β-emitting anti-CD20 mAbs ^90^Y-labeled ibritumomab tiuxetan (Zevalin^®^; Spectrum pharmaceuticals, USA), and ^131^I-labeled tositumomab (Bexxar^®^; GlaxoSmithKline LLC, Delaware, USA), are currently approved treatment options for patients with CD20-expressing B-cell non-Hodkin lymphoma (NHL).

Patients selected for CD20-directed RIT have often already been treated with several cycles of the anti-CD20 antibody rituximab, which may result in a selection of tumor cells with reduced CD20 expression and thus less effect of subsequent anti-CD20 treatments [1-3]. In addition, presence of rituximab in the blood might reduce tumor cell targeting by blocking the antigen and impair the clinical efficacy of CD20-directed RIT [4]. Thus, an improved approach to lymphoma treatment might be to target other antigens than CD20 after some cycles of rituximab treatment. During B-cell development, CD37 is expressed in cells progressing from pre-B to peripheral mature B-cell stages and is absent on terminal differentiation to plasma cells [5]. CD37 internalizes, but has modest shedding in transformed B-cells expressing the antigen [6,7]. Therefore CD37 represents a valuable therapeutic target for malignancies derived from peripheral mature B-cells, such as B-cell chronic lymphocytic leukemia (CLL), hairy-cell leukemia (HCL) and NHL. 

RIT with CD37 as target has been explored previously using a ^131^I-labeled murine monoclonal antibody (MB-1) against CD37 both in a mouse model and in the clinic [8-13]. CD37 antibodies were compared with CD20 antibodies and a higher grade of internalization and degradation of ^131^I-labeled radioimmunoconjugate (RIC) was found for CD37 than for CD20 [13]. Despite the promising clinical responses observed in these early clinical studies, CD20 was deemed more suitable as target than CD37. Therefore, CD20 was chosen as the target antigen for further development of a commercially available RIC. No subsequent efforts have been made to target CD37 with RICs. 

In the early studies of CD37-directed RIT the chloramine T method of ^131^I-labeling was used [13]. ^131^I labeled antibodies have a tendency to cause cellular release of low molecular weight ^131^I catabolites when antigen-antibody complex is internalized [6,7]. The same cellular release of ^131^I has been shown with CD22 antibodies, which also are internalized [14]. However, metallic radionuclides labeled to antibodies with chelators will be better retained inside the cells [15].

The metallic beta-emitter ^177^Lu (T_1/2_ = 6.7 days) has been successfully used in several clinical trials [16-20]. It can be produced by direct neutron activation of ^176^Lu, or via beta decay of reactor-produced ^177^Yb and it is available in GMP quality [21,22]. ^177^Lu might also be used in RIT of relatively small sized tumors because the energy of the beta particle is relatively low, resulting in shorter range in tissue compared to other beta-emitters used for RIT [22].

In an effort to re-evaluate and improve RIT against CD37 we have developed a new radioimmunoconjugate that targets NHL. It is based on ^177^Lu, the backbone substituted chelator p-SCN-Bn-DOTA (tetraxetan) and the anti-CD37 antibody tetulomab (HH1) that was originally developed at the Norwegian Radium Hospital [23]. 

This paper assesses the biodistributions of ^177^Lu-tetraxetan-tetuomab (^177^Lu-tetulomab) in mice with and without tumor xenografts. It includes biodistributions with different protein dosages since this parameter has been reported to influence biodistributions [12,24]. Biodistributions of ^177^Lu-tetulomab and ^177^Lu-rituximab were compared. Finally, absorbed radiation doses for mice and extrapolations to humans were estimated and compared with current dose estimates for clinically approved Zevalin therapy. 

## MATERIALS AND METHODS

### Labeling and Quality Control of Antibodies with ^177^Lu

The antibodies tetulomab and rituximab were first labeled with p-SCN-Bn-DOTA dissolved in 0.005M HCl. The molar ratio of p-SCN-Bn-DOTA to antibody was 6:1. The reaction was pH-adjusted to 8.5 ± 0.2 using carbonate buffer. After 45 minutes of incubation at 37 ^o^C the reaction was stopped by the addition of 50 µl of 0.2 M glycine solution/mg of Ab. To remove free chelator the conjugated antibody was washed by diluting the antibody conjugate 1:10 with 0.9 % NaCl and up-concentrated by centrifugation with AMICON-30 centrifuge tubes (Millipore, Cork, Ireland). The procedure was repeated 4-5 times. Before labeling with 120-220 MBq ^177^Lu (Perkin Elmer, Boston, Ma, USA), 1 mg of antibody conjugated to tetraxetan was pH-adjusted to 5.3 ± 0.3 using 0.25 M Ammonium Acetate buffer. The reaction was incubated for 45 minutes at 37^o^C. 

The radiochemical purity (RCP) of the conjugate was evaluated using instant thin layer chromatography (ITLC). If RCP was below 95% the conjugate was purified using Econo-Pac 10 DG columns (Bio-rad Laboratories, California, USA). The immunoreactive fractions (IRFs) of the RICs were measured using Daudi lymphoma cells and a modified Lindmo method [25]. Cell concentrations of 10, 50, 100 and 200 million cells/ml were used. The IRFs of all RICs used in the experiments were between 50 % and 75 %.

### Animals

Institutionally bred male and female Balb/C nu/nu (NCR) mice that were around 16 weeks old and had body weights in the range of 18 to 35.5 g at the start of the experiment were used, except in the free ^177^Lu biodistribution experiment where the mice were around 6 months old. The animals were maintained under pathogen-free conditions, and food and water were supplied ad libitum. All procedures and experiments involving animals in this study were approved by the National Animal Research Authority and carried out according to the European Convention for the Protection of Vertebrates Used for Scientific Purposes. Mice were anesthetized with 70-100 μl Tiletamin-Zolazepam mix (Virbac, Carros Cedex, France) diluted 1:5 with sterile water and given subcutaneously before implantation with 2x2x2 mm pieces of Daudi lymphoma tumor tissue.

### Biodistribution Experiments

Table **1** gives an overview of the biodistribution experiments. Biodistributions of ^177^Lu- tetulomab and rituximab were determined in female BALB/c-nude (nu/nu) mice with Daudi xenografts with size 32-256 mm^3^ at the start of the study as well as with male BALB/c nude (nu/nu) mice without tumor xenografts. In addition, the effect of protein dosage of 1 mg/kg, 0.1 mg/kg and 0.01 mg/kg on the biodistribution of ^177^Lu-tetulomab was investigated. The preparations were administered by tail vein injection of 100 μl solution to each animal. An approximate activity of 500 kBq was injected (per mouse) for the two RICs. Four to six animals were used per time point.

Biodistribution experiments of free ^177^Lu were performed by injecting a mean activity of 0.86 MBq of ^177^LuCl_3_ per mouse. The concentration of the injection solution was around 50 MBq/ml, diluted in 0.9 % NaCl % and with 0.002 mg/ml of tri-sodium citrate to avoid formation of aggregates. The mice in this experiment were between 6 and 7 months old. 

Autopsies were performed after cervical dislocation at various time points after injection. The weight of each tissue sample was determined, and the amount of ^177^Lu was measured by a calibrated gamma detector (Cobra II auto-gamma detector, Packard Instrument Company, Meriden, CT, USA). Samples of the injectates were used as references in the measurement procedures. The decay corrected percentages of the injected dose per gram tissue (%ID/g) and the non-decay corrected activity per gram tissue (specific activity) were calculated for each time point.

Biological half-life in blood was obtained by fitting a biexponential function to the decay-corrected %ID/g biodistribution data of mice with xenografts. 

### Dosimetry

#### Dosimetry in Mice

The absorbed radiation doses from ^177^Lu-tetulomab and ^177^Lu-rituximab in mice were calculated by multiplying the area under the specific activity versus time curves, normalized to an injection of 40 MBq/kg, by the mean energy of the β-particles, Auger- and conversion electrons of 0.1473 MeV [26]. The area under the specific activity versus time curve was calculated by the trapezoidal rule [27]. The activity at t = 0 was estimated to be zero in all organs except in blood, which was estimated to contain 100 % of the total injected activity. The absorbed radiation doses were adjusted for self-radiation and cross organ radiation by multiplying with a self-radiation factor and a cross-radiation factor developed in reference [28]. For blood and brain 1 was used as self-radiation factor and 0 as cross-radiation factor to and from all other organs. Lymph node was regarded as equivalent to a 25 mg tumor, for which cross and self-radiation factors were calculated in reference [28]. The absorbed fractions for tumor were selected according to the average tumor mass found in ^177^Lu-tetulomab and ^177^Lu-rituximab groups which was 0.3 ± 1.4 g and 0.10 ± 0.08 g respectively.

Dose rate for each organ and for each time point was estimated by multiplying the specific activity by the mean energy of the β-particles, Auger and conversion electrons. The calculation did not take into account cross radiation between organs. 

#### Extrapolation to Humans

The dose extrapolation to humans involved the scaling of the biodistributions and the subsequent calculation of the absorbed radiation dose from the scaled biodistributions. The biodistribution scaling was performed by two alternative methods. Method 1 was based on the assumption that % Injected Dose in humans in each organ (%ID_h) is the same as the %ID in mice for the same organ (%ID_m) [29]. Method 2 considered a relative mass scaling where the specific activity in a certain human organ is equal to the specific activity in the same mouse organ multiplied by the ratio of the body mass of human and mouse [31, 32]. Cumulated activities were calculated using the same method as used for mouse dosimetry calculations. The dose calculation was done for a selected group of organs following the MIRD scheme [30]. The S-values for ^177^Lu for adult male were extracted from RADAR website [31]. Some special considerations were used due to the differences between the organs/tissues used in the MIRD scheme and the organs measured in our biodistribution experiments:

1. MIRD divides the large intestine (both target and source organ) into lower and upper large intestine (LLI and ULI respectively). The measurements were done to the complete large intestine. Therefore, it was assumed that half the measured activity was localized in each section.
2. MIRD divides the heart contribution as source organ into heart wall and heart content. The measurements were done on heart with contents. It was assumed that 10 % of the measured activity was associated to the heart wall and 90 % to the heart contents [32].
3. It was assumed that femur consisted of 33 % trabecular and 67 % cortical bone and that 50 % of the activity in each bone type was located on the surface and 50 % in the volume [33].
4. The weights of the human organs were extracted from [34]. In the case of femur, the specific activity was multiplied by the mass of skeleton in human (10,000 g) to account for activity in bone.
5. The dose to red marrow was calculated by multiplying the average bone dose with 1.7, according to [35]. The factor 1.7 is based on clinical data with a ^177^Lu-labeled monoclonal antibody against prostate cancer, which is expected to have a similar nonspecific biodistribution as ^177^Lu-tetulomab.

## RESULTS

### Biodistribution of ^177^Lu-Tetulomab, ^177^Lu-Rituximab and ^177^LuCl_3_

Biodistributions of ^177^Lu-tetulomab, ^177^Lu-rituximab and ^177^LuCl_3_ were determined in nude mice without tumor xenografts (Table **2**). There were no major differences between the biodistributions of ^177^Lu-tetulomab and ^177^Lu-rituximab and there were no signs of redistribution of nuclide from/to any organs after the initial uptake of the radioimmunoconjugates. The %ID/g in liver and spleen for ^177^Lu-tetulomab was twice that of free ^177^Lu (^177^LuCl_3_). The opposite was found for kidneys. The most significant difference between ^177^Lu bound to antibodies and free ^177^Lu was the difference in uptake in femur and skull for free ^177^Lu. Activity concentration of free ^177^Lu in skull and femur was around 5 and 10 times higher than that of ^177^Lu-tetulomab and ^177^Lu-rituximab. The data showed that the radioimmunoconjugates had a high stability *in-vivo*.

**Fig. (1):**
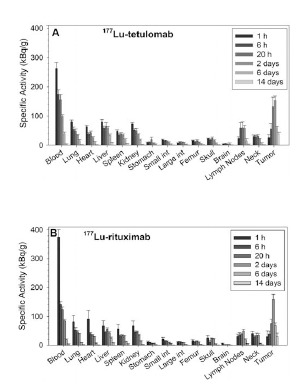
Biodistribution (kBq/g) of ^177^Lu-tetulomab (A) and ^177^Lurituximab (B) in female nude mice with Daudi tumor xenografts. The data were normalized to an injected activity of 1 MBq per mouse (40 MBq/kg). Error bars correspond to standard error.

Biodistributions of ^177^Lu-tetulomab and ^177^Lu-rituximab were also determined in nude mice with Daudi tumor xenografts (Fig. **1**). Both ^177^Lu-tetulomab and ^177^Lu-rituximab were rapidly taken up in most normal tissues and had a slower uptake in tumor. Tumour uptake increased up to 2 days after injection with a maximum between 2 and 6 days, while activity levels in blood and normal tissues cleared significantly during the first few days. The uptake in tumor was higher than in other tissues. The Daudi xenografts doubled in size in 2.4 ± 0.5 days. Thus, the decrease in tumor specific activity observed after 2 days might be due to increase in tumor weight and not only wash out of RICs. In general, the biodistributions for mice with and without xenografts were similar, with a tendency to lower uptake in normal tissues in the former. Tumor/Blood ratio was around 1.5 one day after injection and increased up to around 10 after 14 days. The lowest tumor to normal tissue (T/NT) ratio was found for liver, being around 2 one day after injection and around 5 after 14 days. T/NT ratio in spleen increased up to 6, 14 days after injection. This generally high T/NT ratio indicates that the half-life of ^177^Lu (6.7 days) is suitable to allow a favorable uptake in tumor compared to normal tissues.

The biological half-life of excretion of ^177^Lu-tetulomab from blood was estimated through biexponential fittings to the biodistributions in mice with xenografts (Table **3**). The results from the fittings were similar for both radioimmunoconjugates, with slightly longer half-lives in blood for ^177^Lu-tetulomab. 

### Effect of Protein Dose

Table **4** shows the biodistribution of ^177^Lu-tetulomab in nude mice with xenografts for three different protein dosages (1, 0.1 and 0.01 mg/kg). ^177^Lu-tetulomab was injected intravenously into nude mice carrying Daudi human lymphoma xenografts. Animals were sacrificed after 1 and 5 days and normal tissue and tumor activity was measured. The biodistributions for the different antibody dosages were not significantly different from each other (p = 0,56 for 1 vs. 0.1 mg/kg, p = 0.17 for 1 vs. 0.01 and p = 0.06 for 0.1 vs- 0.01 mg/kg, Chi square test) even though the amount of antibody injected differed by a factor of 100. Some individual tissues, such as blood, lungs, liver, brain and thyroid, however, showed significantly different uptake among the different antibody dosages (p < 0.05, t-test, Table **4**). Tumor uptake tended to be slightly higher for the 0.1 mg/kg dosage though tumor/blood ratio was similar for all the biodistributions, with lowest values found for the 1 mg/kg dosage. 

### Dosimetry

**Fig. (2):**
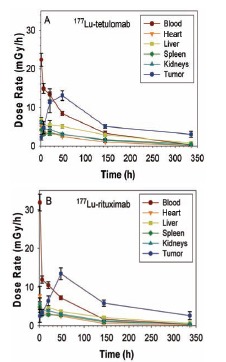
Dose rate (mGy/h) for key organs estimated from biodistributions of ^177^Lu-tetulomab (**A**) and ^177^Lu-rituximab (**B**) in nude mice with Daudi xenografts. The data were normalized to an injected activity of 1 MBq per mouse (40 MBq/kg). Error bars correspond to standard error.

Dose rate in blood decayed exponentially from a value of 22 mGy/h (for ^177^Lu-tetulomab) and 32 mGy/h (for ^177^Lu-rituximab) to around 0.001 mGy/h 14 days after injection (Fig. **2**). Dose rates in tumor reached maximum values at some point between 2 and 6 days and decayed exponentially up to around 0.03 mGy/h 14 days after injection. For the rest of the organs the dose rate was between 0.8 and 0.04 mGy/h during the first 6 hours and decayed to values between 0.007 and 0.003 mGy/h 14 days after treatment. Two weeks after injection the dose rate in tumor was 5 to 20 times higher than in normal tissues.

**Fig. (3):**
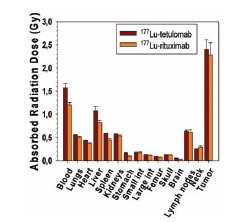
Dose (Gy) to normal organs for mice with xenografts injected with ^177^Lu-tetulomab or ^177^Lu-rituximab. The data were normalized to an injected activity of 1 MBq per mouse (40 MBq/kg). Error bars correspond to standard error.

The absorbed radiation doses to normal tissues for both antibodies were below 1 Gy except for blood, when the injected activity was normalized to 1 MBq/mouse, which is equivalent to 40 MBq/kg for a 25 g mouse (Fig. **3**). The dose to tumor in mice with Daudi xenografts was 2.3 Gy for ^177^Lu-rituximab and 2.4 Gy for ^177^Lu-tetulomab for an injected activity of 40 MBq/kg. The tumor to normal tissue ratios were in general similar for both antibodies (data not shown). 

### Extrapolation to Humans

An extrapolation of the absorbed radiation doses from mouse to human was done assuming an injected activity of 2.8 GBq (40 MBq/kg) and a patient weight of 70 kg (Table **5**). The extrapolations from the last time point of the *activity vs. time* curves to infinity represented between 2 and 11 % of the total area under the curve. The data was compared with clinical dosimetry data of ^90^Y-tiuexetan-ibritumomab (15 MBq/kg) from Fisher *et al.* [39] and of ^177^Lu-cG250 (2,405 GBq/m^2^) from Stillebroer *et al.* [36], which is equivalent to approximately 60 MBq/kg for a patient with 1.79 m^2^ surface area and 70 kg bodyweight. 

All key organs treated with ^177^Lu-tetulomab had lower absorbed doses than those found by Fisher *et al.* [39] where no serious adverse effects were observed in the patients. For all organs the absorbed doses of ^177^Lu-tetulomab were well below the 15 Gy limit suggested by Winter *et al.* (2009) for ^90^Y-ibritumomab [37]. Furthermore, the absorbed doses were lower for both 40 and 60 MBq/kg ^177^Lu-tetulomab than for 2.4 GBq/m2 (60 MBq/kg) ^177^Lu-cG250, except for the dose to red marrow. Different methods were, however, used to estimate the dose to red marrow in the two studies.

## DISCUSSION

Despite being underutilized, the introduction of RIT against NHL has been clinically successful. One hurdle to a widespread use of RIT is that current available products compete with the market leading immunotherapy (rituximab) for the same antigen. A new RIC for NHL, ^177^Lu-labeled anti-CD37 antibody tetulomab has been developed. The biodistribution of ^177^Lu-tetulomab was similar to the biodistribution of ^177^Lu-rituximab in mice with and without xenografts. There were no signs of redistribution of nuclide from/to any organs after initial uptake. The low uptake in bone compared with the biodistribution of free ^177^Lu (^177^LuCl_3_) showed that the RICs were highly stable *in-vivo*. The absorbed radiation dose to tumor in mice with Daudi xenografts was 2.3 Gy for ^177^Lu-rituximab and 2.4 Gy for ^177^Lu-tetulomab for an injected activity of 40 MBq/kg. The absorbed radiation doses for 40 MBq/kg ^177^Lu-tetulomab, when extrapolated to humans, were lower than for 15 MBq/kg ^90^Y-ibritumomab. It should be noted that tetulomab internalizes to a greater extent than rituximab and this could cause additional cytotoxicity to targeted cells. This difference is not accounted for by conventional dosimetry.

Free ^177^Lu accumulated mainly in bone. Breeman *et al.* [38] found that rats injected with ^177^LuCl_3_ had an uptake in femur of 5.1 %ID/g after 24 h, while it was 10 % ID/g in our study. The uptake in bone was explained by the similarity between the ions ^177^Lu^+3^ and Ca^+2^ that are involved in hydroxiapatite formation. Muller *et al.* [39] found that the biological half-lives of ^177^Lu was around 5 days for soft tissues while it was 50 days for skeleton. Given the low concentration of activity found in femur and skull in the biodistribution studies of ^177^Lu-tetulomab, ^177^Lu does not seem to detach from the tetraxetan chelator to a significant degree after injection.

The maximum tumor uptake between 2 and 6 days after injection indicate that a radioisotope with a half-life longer than 3 days will be more suitable to obtain a favorable tumor to normal tissue ratio than a radioisotope with a shorter half-life. For this reason ^177^Lu (t_1/2_ = 6.7 days), may be more suitable than ^90^Y (t_1/2_ = 2.67 days) for RIT. There was no significant reduction in the tumor cell uptake of ^177^Lu-tetulomab *in vitro* and *in-vivo* after pretreatment with rituximab (unpublished data), which indicates that pretreatment is a relevant strategy for the clinic. This suggests that ^177^Lu-tetulomab can be used both in rituximab treated and rituximab naïve patients.

The antibody dosage may influence the effectiveness of a RIC. Press *et al.* (1989) [12] found in a phase 1 study using three different protein dosages (0.5 mg/kg, 2.5 mg/kg and 10 mg/kg) that the most favorable biodistribution (best tumor retention and localization) was found for the highest dosage. A too low specific activity might limit the number of radioactive atoms able to bind per cell and a too high specific activity might result in a modified biodistribution because of some low level antigen expression in normal tissues could trap a relatively larger fraction of the product when very low amounts of antibodies are used. To address the case of tetulomab, biodistributions and tumor uptake of ^177^Lu-tetulomab were measured in nude mice carrying Daudi human lymphoma xenografts. The results showed that a variation of the antibody dosage with a factor of 100 (between 0.01 and 1 mg/kg) did not significantly change the biodistribution of ^177^Lu-tetulomab. 

The absorbed radiation dose in tumor was considerably higher than in normal tissue and the absorbed radiation doses to normal tissues were reduced in mice with tumor xenografts compared to mice without xenografts.

The major toxicity of RICs for treatment of refractory or relapsed NHL (Zevalin or Bexxar), and ^177^Lu-cG250 is myelosuppression and it is expected that this will be the case also for ^177^Lu-tetulomab. Therefore, it is promising that the estimated absorbed dose to red marrow in humans from a clinically relevant dosage of 40 MBq/kg was considerably lower than the dose limit of 3 Gy reported for the Zevalin (^90^Y-ibritumomab) protocol [40]. However, it is important to keep in mind that the results from our extrapolation to humans from mice data are crude estimates. 

## CONCLUSION

The anti-CD37 antibody tetulomab (HH1) was evaluated as carrier for ^177^Lu. Biodistribution of ^177^Lu-tetulomab was similar to that of ^177^Lu-rituximab in mice and there was a similar xenograft targeting of the two conjugates in Balb C nude mice. The low uptake in bone compared with the biodistribution of free ^177^Lu (^177^LuCl_3_) showed that the radioimmunoconjugates were highly stable *in-vivo*. The half-life of ^177^Lu allows significant tumor to normal tissue ratios being reached before the major radioactivity has decayed. Dosimetric estimates were performed and extrapolated to the human situation and indicated an acceptable bone marrow radiation dose for a clinical relevant dosage of radioimmunoconjugate. In summary the current study shows that ^177^Lu-tetulomab which targets the CD37 antigen is a promising candidate for radioimmunoconjugate therapy against NHL. 

## Figures and Tables

**Table 1.** Overview of Biodistribution Experiments

Experiment	Conjugates	Injected Act. (MBq/kg)	Protein Dosage (mg/kg)	Spec. Act. (MBq/mg)	No. Time Points	No. Mice / Time Point	Mice Sex
Mice without tumor	^177^Lu-tetulomab	20	0.17 - 0.8	25 to 120	4	4 - 5	Male
	^177^Lu-rituximab						
Mice with tumor	^177^Lu- tetulomab	20	0.17 - 0.8	25 to 120	6	4 - 5	Female
	^177^Lu- rituximab						
Protein Dose	^177^Lu- tetulomab	13.7	1	13.7	2	4	Female
			0.1	137			
			0.01	137			
Free ^177^Lu		43			4	6	Female

**Table 2.** Biodistribution (%ID/g) of ^177^Lu-Rituximab, ^177^Lu-Tetulomab and ^177^LuCl_3_ in nude mice without xenografts.

	^177^Lu-rituximab				^177^Lu-tetulomab				^177^LuCl_3_			
Time point (h)	1	24	48	144	1	24	48	144	1	24	48	144
Blood	22 ± 5	14 ± 3	9 ± 4	7 ± 3	26 ± 10	14 ± 2	14 ± 3	15 ± 6	11.0 ± 0.7	0.08 ± 0.01	0.024 ± 0.003	0.010 ± 0.001
Lungs	6 ± 1	6 ± 2	4 ± 1	3 ± 2	7 ± 4	5 ± 1	4 ± 1	5 ± 2	6.3 ± 0.3	1.6 ± 0.1	1.3 ± 0.1	1.2 ± 0.1
Liver	7 ± 5	5 ± 2	5 ± 2	3.4 ± 0.9	11 ± 8	7 ± 5	4.5 ± 0.7	5 ± 2	3.9 ± 0.2	3.8 ± 0.1	3.3 ± 0.1	2.18 ± 0.06
Spleen	7 ± 2	5 ± 3	4 ± 2	5 ± 2	6 ± 3	3.5 ± 0.8	5 ± 1	7 ± 4	2.5 ± 0.2	1.2 ± 0.4	0.9 ± 0.1	1.02 ± 0.04
Kidneys	7 ± 2	5 ± 2	4 ± 1	3 ± 1	7 ± 2	4.0 ± 0.9	4.2 ± 0.9	4 ± 2	7 ± 1	11.1 ± 0.7	7.9 ± 0.7	5.8 ± 0.2
Femur	2.4 ± 0.9	2 ± 1	1.7 ± 0.7	2 ± 2	2.3 ± 0.7	3 ± 2	2 ± 1	3 ± 1	6.4 ± 0.6	10 ± 1	13 ± 1	13 ± 1
Skull	3 ± 1	4 ± 1	3 ± 1	2 ± 2	3 ± 1	4 ± 2	3 ± 2	3 ± 2	10.1 ± 0.5	26 ± 5	32 ± 4	32 ± 5
Lymph Nodes	3 ± 1	7 ± 5	4.9 ± 0.6	2.4 ± 0.7	2 ± 1	8 ± 3	5 ± 1	6 ± 4	6 ± 2	1.4 ± 0.2	1.9 ± 0.4	1.2 ± 0.2

**Table 3.** Plasma Clearance Kinetics of ^177^Lu-tetulomab and ^177^Lu-rituximab in Nude Mice with Daudi xenografts

Pharmacokinetic Parameter	^177^Lu-tetulomab	^177^Lu-rituximab
*T^Bio^*_½_**(monoexponential^a^)**	59 ± 12 h	23 ± 6 h
*T^Eff^*_½_**(monoexponential)^b^**	43 ± 11 h	20 ± 5 h
**α (biexponential^c^)**	2.3 ± 2 h	0.7 ± 1.4 h
**β (biexponential^c^)**	101 ± 29 h	74 ± 15 h

a y=a×e−lnz×tT12Bio

b 1T12Eff=1T12Biol+1T12Phys

c $y=a\times e^{-\frac{\ln\left(z\right)\times t}{\alpha }}+b\times e^{-\frac{\ln\left(z\right)\times t}{\beta }}$

**Table 4.** Biodistribution (%ID/g) of Different Protein Dosages of ^177^Lu-tetulomab in Nude Mice with Daudi Xenografts

Protein Dosage	1 mg/kg		0.1 mg/kg		0.01 mg/kg	
Time point (h)	22	120	22	120	22	120
Blood	13 ± 1^b^	5 ± 2	13.2 ± 0.9	7 ± 2	8 ± 2^a^	3.8 ± 0.3
Lungs	4.7 ± 0.5^a^	2.1 ± 0.6	6.7 ± 0.5	3.4 ± 0.9	3.3 ± 0.3^b^	1.9 ± 0.3
Liver	5.6 ± 0.7	4.4 ± 0.7	6.9 ± 0.5	6.4 ± 0.6	4.3 ± 0.8^a^	3.9 ± 0.4^a^
Spleen	2.7 ± 0.5	2.3 ± 0.6	2.8 ± 0.2	3.1 ± 0.5	2.1 ± 0.2^a^	2.1 ± 0.3
Kidneys	4.8 ± 0.8	4 ± 1	3.5 ± 0.2	3.0 ± 0.6	3.6 ± 0.6^a^	1.7 ± 0.2
Femur	1.6 ± 0.2	0.7 ± 0.3	4 ± 3	1.3 ± 0.4	1.1 ± 0.2	0.5 ± 0.1
Skull	2.4 ± 0.3	1.1 ± 0.3	1.1 ± 0.5	1.6 ± 0.5	1.9 ± 0.4	0.8 ± 0.1
Lymph Nodes	16 ± 5	5 ± 2	13 ± 3	9 ± 3	9 ± 2	4.7 ± 0.9
Tumor	11 ± 3	6 ± 2	14 ± 3	11 ± 5	7 ± 1	8 ± 2

a Statistically significant difference when compared with 0.1 mg/kg (p < 0.05, t-test)

b Statistically significant difference when compared with 0.01 mg/kg (p < 0.05, t-test)

**Table 5.** Extrapolation of Absorbed Radiation Dose (Gy) in Mice with Daudi Xenografts to Humans and Comparison with Absorbed Radiation Doses in Patients Treated with ^90^Y-Tiuxetan-ibritumomab and ^177^Lu-cG250

Organ	40 MBq/kg ^177^Lu-tetulomab		15 MBq/kg ^90^Y-tiuexetan-ibritumomab	2.4 GBq/m^2^ ^177^Lu-cG250
**Method**	**%ID^a^**	**MS^b^**	**Clinical Data^c^**	**Clinical Data^d^**
**Lungs**	0.28	0.58	0.80 ± 0.51	2.1 ± 0.6
**Heart**	0.25	0.51	1.13 ± 0.41	3.3 ± 0.7
**Liver**	2.12	1.10	3.82 ± 1.46	5.4 ± 1.1
**Spleen**	1.61	0.66	4.88 ± 2.44	
**Kidneys**	2.00	0.64	2.56 ± 0.64	5.6 ± 1.5
**Stomach**	0.36	0.12	0.40 ± 0.12	
**Small int.**	1.02	0.22	0.40 ± 0.12	
**Large int.**	1.40	0.18	0.40 ± 0.12	
**Femur/Bone**	0.76	0.98	2.25 ± 0.54	
**Red marrow**	1.29^d^	1.67^d^	2.87 ± 0.95	1.5 ± 0.3

a It was assumed that %ID in humans in each organ was the same as the %ID in mice for the same organ.

b Relative mass scaling method, where the specific activity in a certain human organ is equal to the specific activity in the same mouse organ multiplied by the ratio of the body mass of human and mouse.

c Results were given as mean ± standard deviation of absorbed dose per MBq of injected activity in Fischer *et al..* We multiplied these values by 15 MBq/kg and 70 kg bodyweight.

d Stillebroer *et al.* gave the mean absorbed dose to liver and kidney for the maximum tolerated dose (MTD) of 2,405 GBq/m^2^ (60 MBq/kg) administered to 7 patients. In addition, mean values of absorbed dose per MBq for 20 patients treated with 1110 MBq/m^2^ to 2405 MBq/m^2^ were given. The values presented in Table 5 are the result of multiplying the reported values by the MTD.

e Assuming red marrow dose is 1.7 times the average bone dose for ^177^Lu –labeled monoclonal antibodies (Vallabhajosula S, 2005).
